# An Integrated Analysis of Prognostic Signature and Immune Microenvironment in Tongue Squamous Cell Carcinoma

**DOI:** 10.3389/fonc.2022.891716

**Published:** 2022-07-13

**Authors:** Yi Jin, Zhanwang Wang, Weizhi Tang, Muxing Liao, Xiangwei Wu, Hui Wang

**Affiliations:** ^1^ Department of Radiation Oncology, Hunan Cancer Hospital, The Affiliated Cancer Hospital of Xiangya School of Medicine, Central South University, Changsha, China; ^2^ Key Laboratory of Translational Radiation Oncology, Department of Radiation Oncology, Hunan Cancer Hospital, The Affiliated Cancer Hospital of Xiangya School of Medicine, Central South University, Changsha, China; ^3^ Department of Oncology, Third Xiangya Hospital of Central South University, Changsha, China; ^4^ Department of Oncology, Hunan Academy of Chinese Medicine Affiliated Hospital, Changsha, China; ^5^ Department of Oncology, Youxian People’s Hospital, Zhuzhou, China

**Keywords:** tongue squamous cell carcinoma, immune checkpoint inhibitor, WGCNA, immune microenvironment, prognostic signature

## Abstract

Tongue squamous cell carcinoma (TSCC) is a prevalent cancer of the oral cavity. Survival metrics are usually unsatisfactory, even using combined treatment with surgery, radiation, and chemotherapy. Immune checkpoint inhibitors can prolong survival, especially in patients with recurrent or metastatic disease. However, there are few effective biomarkers to provide prognosis and guide immunotherapy. Here, we utilized weighted gene co-expression network analysis to identify the co-expression module and selected the turquoise module for further scrutiny. Gene Ontology and Kyoto Encyclopedia of Genes and Genomes analyses revealed the innate pathways. The findings indicated that cell junction organization, response to topologically incorrect protein, and regulation of cell adhesion pathways may be essential. Eleven crucial predictive genes (*PLXNB1*, *N4BP3*, *KDELR2*, *INTS8*, *PLAU*, *PPFIBP2*, *OAF*, *LMF1*, *IL34*, *ZFP3*, and *MAP7D3*) were used to establish a risk model based on Cox and LASSO analyses of The Cancer Genome Atlas and GSE65858 databases (regarding overall survival). Kaplan–Meier analysis and receiver operating characteristic curve suggested that the risk model had better prognostic effectiveness than other clinical traits. Consensus clustering was used to classify TSCC samples into two groups with significantly different survival rates. ESTIMATE and CIBERSORT were used to display the immune landscape of TSCC and indicate the stromal score; specific types of immune cells, including naïve B cells, plasma cells, CD8 T cells, CD4 memory resting and memory activated T cells, follicular helper T cells, and T regulatory cells, may influence the heterogeneous immune microenvironment in TSCC. To further identify hub genes, we downloaded GEO datasets (GSE41613 and GSE31056) and successfully validated the risk model. Two hub genes (*PLAU* and *PPFIBP2*) were strongly associated with CD4+ and CD8+ T cells and programmed cell death protein 1 (PD1) and PD-ligand 1.

## Introduction

Tongue squamous cell carcinoma (TSCC) is comparatively silent when it progresses from a premalignant state to the malignant stage. It is one of the most common cancers of the oral cavity (1, 2). The delayed appearance of specific symptoms, such as pain, can hinder diagnosis and result in a poor prognosis (3). The 5-year relative survival rate is unsatisfactory, particularly in the advanced stages, even using combined treatments involving surgery, radiation, and chemotherapy (4). There has been renewed enthusiasm for cancer immunotherapy, which is reportedly an effective treatment modality for multiple tumor types, especially head and neck tumors (5). Recent clinical trials have shown that inhibitors of immune checkpoints, including programmed cell death 1 (PD-1) and programmed cell death ligand 1 (PD-L1), may prolong survival of patients with platinum-refractory, recurrent/metastatic stage TSCC (6), or lymph node involvement (7). However, the prognostic value of PD-1/PD-L1 expression remains unclear because only 20% of patients respond to immunotherapy. Currently, the tumor, lymph node, and metastasis (TNM) classification system is widely used. However, it is difficult to accurately predict the clinical outcome of TSCC. One important reason is the disregard of individual differences in genetic and biological behaviors, with the resulting use of uniform treatment for patients with the same clinical and histological features. Immune-related prognostic biomarkers with clinical utility are urgently needed.

Recently, hundreds of tumor biomarkers have been evaluated for their potential prognostic ability in TSCC (8). Rare molecular biomarkers for TSCC have been approved for use in clinical practice, and they improve classical prognostic methods, such as TNM stage identification. It is important to select promising biomarkers that are superior to the current predictive system using a valid systematic analysis. Weighted gene co-expression network analysis (WGCNA) is a novel biological method that helps us understand disease mechanisms based on the correlation between changes in gene expression values and the complex distribution of clinical patterns (9). WGCNA technology has been successfully used in many cancers, including prostate cancer (10), lung cancer (11), and breast cancer (12). Overall, for this multi-factor and multi-stage complex disease, WGCNA may be effective for comprehensively elucidating TSCC genomics.

In this study, we used WGCNA to cluster prognostic genes into different functional groups based on The Cancer Genome Atlas (TCGA) and Gene Expression Omnibus (GEO) databases. We also used ESTIMATE, CIBERSORT, and LASSO regression analyses to further identify their prognostic significance and explore the underlying mechanisms of changes in the tumor immune microenvironment associated with TSCC.

## Materials and Methods

### Raw Data Download

RNA sequencing (fragments per kilobase of transcript per million mapped reads [FPKM]) and clinical information, including age, sex, and TNM stage, were downloaded from publicly available data from TCGA head and neck squamous cell carcinomas and GEO databases. The primary site in these samples was the tongue. One TCGA dataset and three eligible GEO datasets (GSE65858, GSE41613, and GSE31056) provided data for background adjustment and quantified normalization.

### WGCNA Data Processing

The Kaplan–Meier survival package of R was used to perform Cox proportional hazard regression analysis to identify differentially expressed genes (DEGs) linked to overall survival (OS). The Limma R language package was applied to screen DEGs according to the criterion of false discovery rate (FDR) < 0.05 compared with the differences between normal and cancer tissues. Genes with prognostic value and similar cancer association were included in co-expression analysis.

### Weighted Co-expression Network Construction

Screened DEGs were prepared for further scale-free network construction and were inputted to test their availability based on the WGCNA R package (9). First, the appropriate soft threshold power β was determined *via* power calculation. Six clinical characteristics were incorporated: race, status, M grade, T grade, N grade, and stage. A sample clustering tree that was constructed combined clinical characteristics and used the dynamic tree cut algorithm to detect the modules. Finally, Pearson’s correlation coefficient was used to construct an adjacency matrix to describe the relationship between co-expression modules and clinical factors.

### Identification of Signaling Pathways

Identification of the co-expression module containing similar functional genes may reveal the major signaling pathways in TSCC. We also used the Metascape (http://metascape.org) portal to perform Gene Oncology (GO) analysis with representative enriched terms and to construct a network colored according to identified clusters. Furthermore, the clusterProfiler R package was used to perform Kyoto Encyclopedia of Genes and Genomes (KEGG) pathway analysis.

### Establishment of the Prognostic Risk Model

Cox analysis was performed to calculate significant prognostic genes in TCGA. The genes were validated from the co-expression modules. LASSO Cox regression analyses were then applied to filter core genes and construct the prognostic models according to the risk score as follows:


$$\begin{array}{l} \text{risk score}={\text{Expression}}_{\text{mRNA}1}\times {\text{Coefficient}}_{\text{mRNA}1}+{\text{Expression}}_{\text{mRNA}2}\times \;\\ {\text{Coefficient}}_{\text{mRNA}2}+\ldots {\text{Expression}}_{\text{mRNAn}}\times {\text{Coefficient}}_{\text{mRNAn}} \end{array}$$

Patients were assigned to high-risk (above the median cutoff of risk score) and low-risk groups (below the median cutoff of risk score). Kaplan–Meier survival and receiver operating characteristic (ROC) curve analyses were performed to further evaluate the prediction accuracy. To enhance prediction accuracy and interpretability, we downloaded three GEO cohorts (GSE65858, GSE41613, and GSE31056) as validation sets to select key prognostic modulators.

### Immune Landscape in TSCC

To further understand the correlations among these key prognostic modulators, consensus clustering analysis was performed to classify TSCC samples using the consensus cluster plus package. To explore the immune landscape of TSCC, the “estimate” component of R software was used to calculate tumor purity, stromal score, immune score, and ESTIMATE score of each tongue tumor sample. The CIBERSORT package was then used to accurately determine the composition of immune cells in the large tumor sample data. Significant results (*p* < 0.05) were used in further analysis.

### Cell Culture and Transfection

The Cal-27 human TSCC cell line was obtained from Procell Company (Wuhan, China). Cal-27 cells were maintained in Dulbecco’s Modified Eagle Medium (DMEM; Procell Company) supplemented with 10% fetal bovine serum (FBS; Gibco, Carlsbad, CA, USA) and 1% penicillin-streptomycin liquid (Procell Company). Cal-27 cells were cultured in a humidified atmosphere containing 5% CO_2_ at 37°C. Small interfering RNAs (siRNAs) targeting *plasminogen activator urokinase (PLAU)* and *PPFIA binding protein 2* (*PPFIBP2*) were synthesized by GenePharma (Shanghai, China). Lipofectamine 2000 (Invitrogen, Carlsbad, CA, USA) was used to transfect the siRNAs into Cal-27 cells according to the manufacturer’s protocol. The transfection efficiency was assessed by quantitative reverse transcription polymerase chain reaction (qRT-PCR).

### qRT-PCR

TRIzol reagent (TaKaRa Bio, Dalian, China) was used to extract total RNAs following the manufacturer’s instructions. RNA (1 μg) was used to synthesize cDNA by a reverse transcription kit (RR037A; TaKaRa Bio). qRT-PCR was performed using the TB Green Premix Ex Taq Kit (RR820A; TaKaRa Bio). The 2^−ΔΔCt^ method was used to calculate the silencing efficiency of the siRNAs. Glyceraldehyde-3-phosphate dehydrogenase (GAPDH) was used as an internal control. The primer sequences are summarized in **Supplementary Table S1**.

### Cell Counting kit-8 (CCK-8) Assay

The proliferation of Cal-27 cells was examined using the CCK-8 kit (Biosharp, Hefei, Anhui, China). Cal-27 cells were transfected with negative control or siRNAs targeting *PLAU* and *PPFIBP2*. CCK-8 (10 μl) was added to cells and incubated at 37°C for the indicated times. The optical density at 450 nm (OD_450_) was measured using a microplate spectrophotometer.

### 5-Ethynyl-2’-deoxyuridine (EdU) Assay

The BeyoClick™ EdU-488 Cell Proliferation Kit (Beyotime, Shanghai, China) was used to stain cells to determine proliferation. Briefly, Cal-27 cells (1×10^5^ cells/well) were seeded in 6-well plates, transfected with different siRNAs, and cultured in an incubator. Cells were incubated with EdU for 2 h after 72 h of transfection, fixed with 1 ml of 4% paraformaldehyde for 20 min, and permeabilized with 0.3% Triton X-100 (Beyotime) for 20 min. Subsequently, the cells were incubated with 500 µl of the click reaction mixture for 30 min at room temperature in the dark, washed three times, and incubated with Hoechst stain for 10 min.

### Clone Formation Assay

Cal-27 cells (4×10^5^ per well) in 6-well plates were transfected with different siRNAs. The cells were collected by trypsinization after 48 h of transfection. Cells (*n* = 1,000) were seeded in 6-well plates and incubated at 37°C for 2–3 weeks. Cell colonies were fixed with 4% paraformaldehyde for 30 min and stained with crystal violet solution (G1073; Solarbio, Beijing, China) for another 30 min. Fixed and stained cells that added up to 50 was counted as a clone.

### Transwell Assay of Cell Migration and Invasions

Transwell chambers (3422; Costar, Corning, NY, USA) that were uncoated or coated with Matrigel Matrix (356234, Corning, USA) were used to detect the migration and invasion of Cal-27 cells. After 24 h of transfection, Cal-27 cells were collected by trypsinization. Cells (at a density of 5×10^5^/ml) were diluted in serum-free DMEM. The upper Transwell chamber was seeded with 200 μl of cell suspension (1×10^5^ cells/well). The lower chamber contained 600 μl of DMEM containing 20% FBS. The Cal-27 cells were incubated for 24 h. The cells that invaded across the chamber membrane were fixed and stained with crystal violet stain solution (G1073; Solarbio) for 30 min. Five randomly photographed fields were selected and the invading cells were counted using an inverted microscope.

### Statistical Analyses

Analyses were performed using R language software. Cox proportional hazards regression analysis was used to select the independent prognostic genes linked to OS. Kaplan–Meier curves were used to compare the clinical outcomes of the subgroups. The Wilcoxon rank-sum test was used to compare the ESTIMATE algorithm. In all analyses, statistical *p*-values were bilateral, and statistical significance was set at *p* < 0.05.

## Results

### Construction of Weighted Co-expression Network

**Figure 1:**
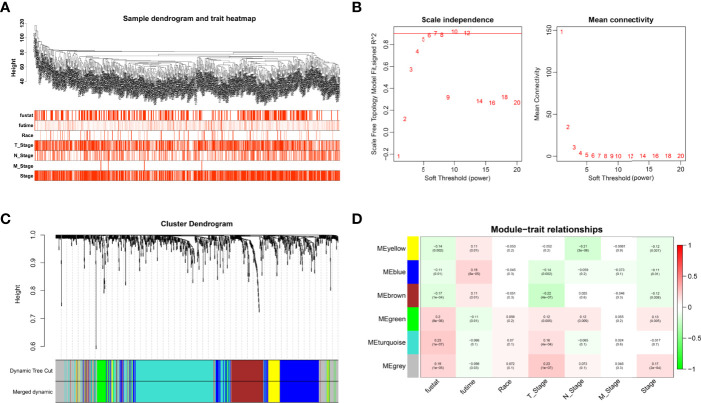
Weighted co-expression network construction. **(A)** Dendrogram and trait heatmap for TSCC patients. **(B)** Network topology for different soft-thresholding powers. **(C)** A cluster dendrogram based on the dynamic tree cut algorithm. **(D)** Module–trait relationship visualization of co-expression modules and clinical features.

Cox analysis identified 1,391 DEGs as protective biomarkers and 1,346 DEGs as risk factors associated with poor OS (**Supplementary Table 1**). Analysis using the Limma package revealed that 6,620 molecules prevented cancer from developing and 7,306 molecules might induce the formation of carcinoma (**Supplementary Table 2**). We further identified 1,007 DEGs associated with TSCC. These were used to construct a weighted co-expression network (**Supplementary Table 3**). To filter the DEGs, clinical traits, including status, race, and M, N, and T stages, were merged. The dendrogram and trait heatmap for these patients are shown in **Figure 1A**. The data show that the samples can be divided into multiple groups according to differences in clinical features.

**Figure 2:**
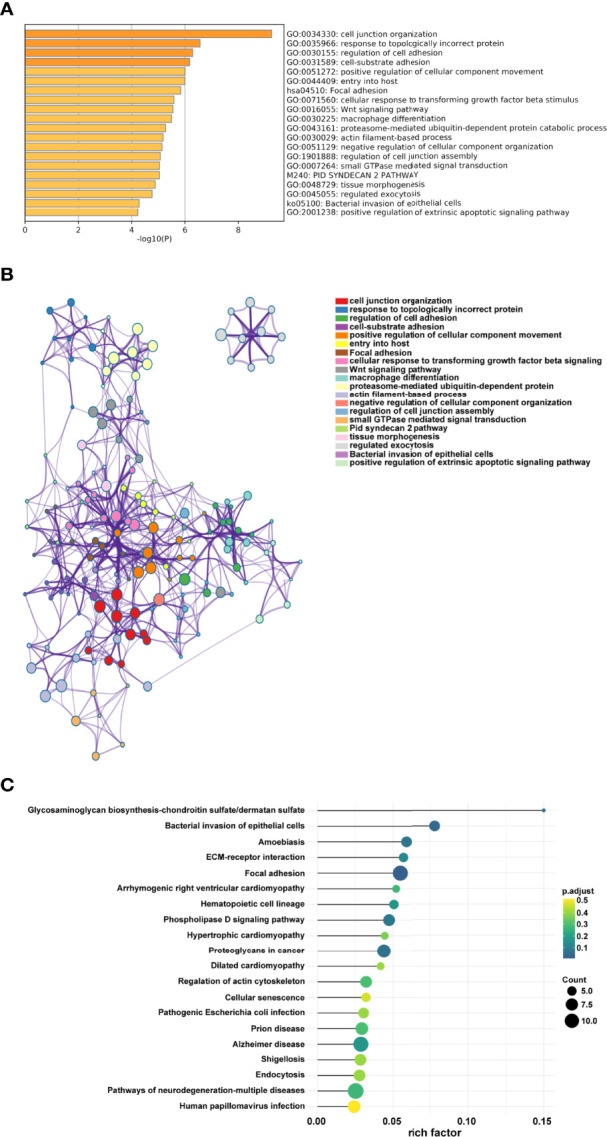
Pathway function analysis. **(A)** Representative enriched terms of GO function. **(B)** The network colored by cluster ID of GO functions. **(C)** Representative enriched terms of KEGG analysis.

The appropriate soft threshold power *β* was determined as six for subsequent adjacency calculations (**Figure 1B**). We identified gene co-expression modules based on the dynamic tree cut algorithm, as shown in **Figure 1C**. Finally, we evaluated and visualized the relationship between co-expression modules and clinical features, which indicated that the turquoise, green, and gray modules were significantly associated with the survival status (**Figure 1D**). Owing to the limited predictive value of TNM stage, we chose the turquoise module, which contained 244 genes, for further investigation. Applying Metascape, we conducted the GO function with representative enriched terms (**Figure 2A**) and a network colored by cluster ID (**Figure 2B**), which showed that the pathways of cell junction organization, response to topologically incorrect proteins, and regulation of cell adhesion may play an essential role in the turquoise module. KEGG analysis showed that focal adhesion pathways were enriched during the development of TSCC (**Figure 2C**).

### Construction and Evaluation of Prognostic Signature

**Figure 3:**
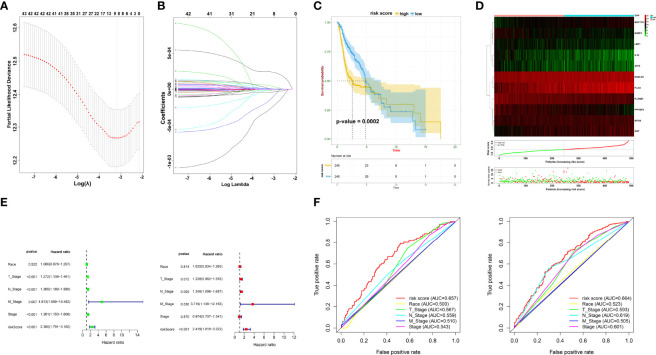
Prognostic signature construction. **(A, B)** Screening of candidate crucial genes from the LASSO Cox regression. **(C)** The Kaplan–Meier plot of high- and low-risk groups. **(D)** Distributions of risk scores, alive/dead status, and expression of crucial prognostic genes. **(E)** Univariate (left) and multivariate Cox analyses (right) of clinical traits. **(F)** ROC curve of risk score and clinical traits in 1 and 3 years.

To accurately filter crucial genes, data from the GSE65858 database were downloaded and prognostic biomarkers were identified using Cox analysis (**Supplementary Table 4**). The results showed that 747 DEGs were correlated with better survival and 1,244 DEGs were associated with poor OS. The collective findings from TCGA, WGCNA, and GSE65858 identified 44 genes as crucial prognostic biomarkers. These were examined as the first step in establishing the risk model (**Supplementary Table 5**). Based on the LASSO Cox regression analysis of the corresponding 44 mRNAs, 11 genes (*PLXNB1*, *N4BP3*, *KDELR2*, *INTS8*, *PLAU*, *PPFIBP2*, *OAF*, *LMF1*, *IL34*, *ZFP3*, and *MAP7D3*) were selected to build the risk model (**Figures 3A, B**, **Supplementary Table 6**). Coefficient_mRNA_ of the risk score is shown in **Supplementary Table 6**. Subsequently, patients were separated into low- or high-risk groups based on the median cutoff of risk score. The high-risk patients had worse OS than those in the low-risk group (*p* = 0.0002) (**Figures 3C, D**). Univariate and multivariate analyses showed that the risk score was an independent prognostic biomarker (**Figure 3E**). The ROC curve showed that the risk score had better prognostic effectiveness than other clinical traits, with an area under the ROC curve of 0.657 and 0.664 at 1 and 3 years, respectively (**Figure 3F**).

### Establishment of Subgroups Based on Prognostic Biomarkers

**Figure 4:**
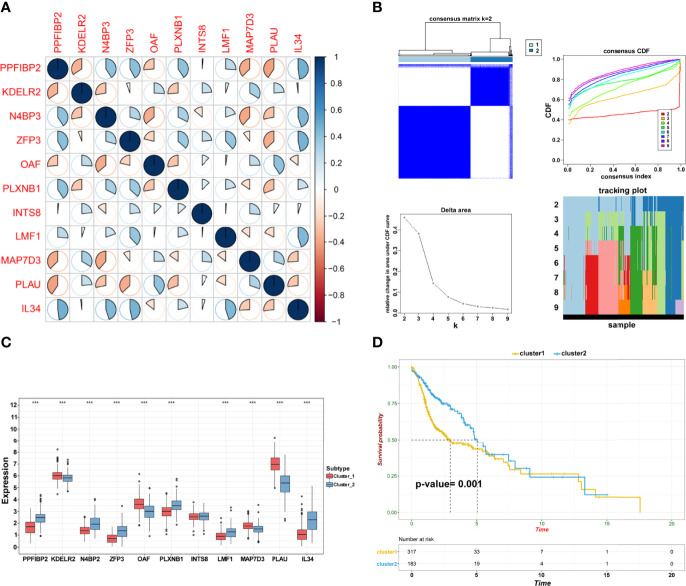
Establishment of subgroups. **(A)** The correlation among crucial candidate genes. **(B)** Consensus clustering and evaluation of samples. **(C)** Differential expression of candidate crucial genes in the two clusters (***p < 0.001; **p < 0.01; *p < 0.05). **(D)** Kaplan–Meier plot of cluster 1 and cluster 2.

Pearson’s correlation analysis was performed to investigate the potential interactions involved in the selected genes and reveal relationships among these prognostic biomarkers (**Figure 4A**). Consensus clustering analysis assigned the TSCC samples into two clusters (**Figure 4B**). The expression pattern of genes in cluster 1 was remarkably different from that in cluster 2 (**Figure 4C**). Interestingly, Kaplan–Meier analysis revealed that the OS between the two clusters was significantly different (*p* = 0.0010; **Figure 4D**). The findings suggest that TSCC is a heterogeneous cancer mediated by specific biomarkers.

### Exploration of Immune Characteristics in TSCC

**Figure 5:**
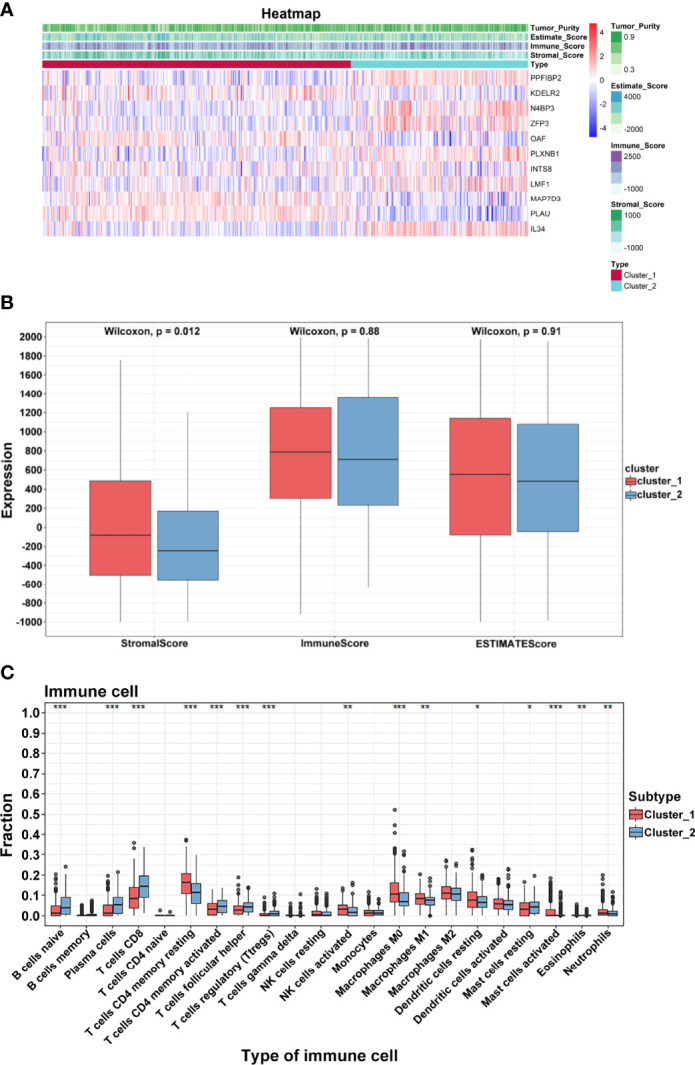
Immune characteristics. **(A)** Heatmap of crucial genes and ESTIMATE scores. **(B)** Differential expression of stromal, immune, and ESTIMATE scores. **(C)** Differential distribution of 22 tumor microenvironment infiltrating cells in two clusters (***p < 0.001; **p < 0.01; *p < 0.05).

TSCC is considered to be immunogenic, in part because of higher levels of tumor-infiltrating lymphocytes. Based on two clusters, a heatmap was drawn to comprehensively describe the immune landscape (**Figure 5A**). The ESTIMATE algorithm was then used to display immune-related scores. The stromal score in cluster 1 was higher than that in cluster 2 (*p* = 0.0002; **Figure 5B**). Results of the CIBERSORT method showed that many immune cells contributed to the alteration of the immune environment, especially naïve B cells, plasma cells, CD8 T cells, CD4 memory resting and memory activated T cells, follicular helper T cells, and T regulatory cells (Tregs) (**Figure 5C**). These results indicated that functional clusters based on these prognostic biomarkers play an important role in deciphering the heterogeneous immune landscape of TSCC.

### Identification of Immune-related Hub Genes

**Figure 6:**
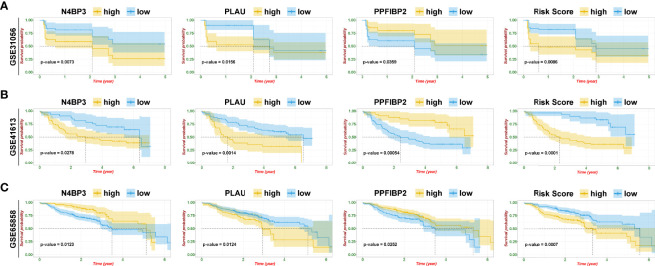
Risk model and validation of hub genes. **(A–C)** Kaplan–Meier plot of hub genes and risk score in GSE31056 **(A)**, GSE41613 **(B)**, and GSE65858 **(C)**.

**Figure 7:**
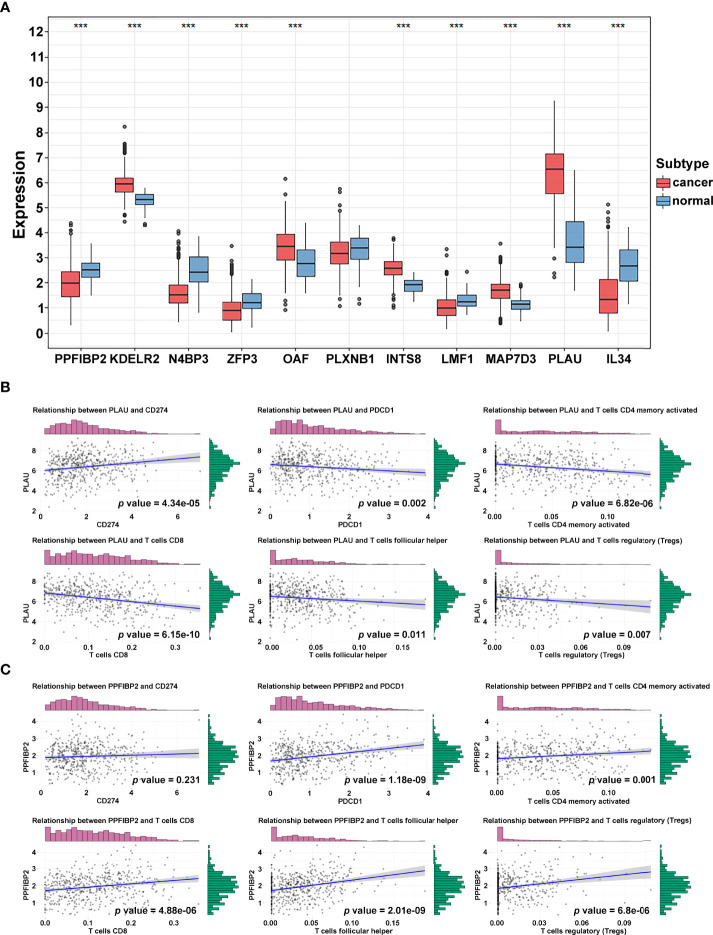
Immune-related hub gene identification. **(A)** Differential expression of crucial genes in cancer and normal tissue (***p < 0.001; **p < 0.01; *p < 0.05). **(B, C)** Correlation of PLAU **(B)** and PPFIBP2 **(C)** and immune biomarkers.

To further identify and verify the predictive ability of the 11 significant mRNAs, we downloaded one GEO (GSE65858) with OS and two GEO (GSE41613 and GSE31056) with progression-free survival (PFS). The risk model and two genes (*PLAU* and *PPFIBP2*) were successfully verified as effective prognostic biomarkers for OS and PFS (**Figures 6A–C**, **Supplementary Figures 1**-**3**). *N4BP3* presented a positive trend in all databases, yet the trend was opposite. Compared to those in normal tissues, the expression level of *PLAU* remarkably increased, and the expression level of *PPFIBP2* noticeably declined. There was no significant change in the expression level of *N4BP3* (**Figure 7A**). Therefore, we investigated the immune environment using these three biomarkers. The results showed that all three hub genes were intimately associated with the expression of PDCD1/PD-1, CD274/PD-L1, and multiple types of T cells (**Figures 7B, C**). Pan-cancer analysis explored differences of *PLAU* and *PPFIBP2* in multiple cancers (**Supplementary Figures 6A** and **7A**), as well as the correlation between immune cells and hub genes. The results indicated that *PLAU* and *PPFIBP2* may influence the immune environment in multiple cancers (**Supplementary Figures 6B** and **7B**).

### Influence of *PLAU* or *PPFIBP2* Depletion on Proliferation, Migration, and Invasion of TSCC Cells

**Figure 8:**
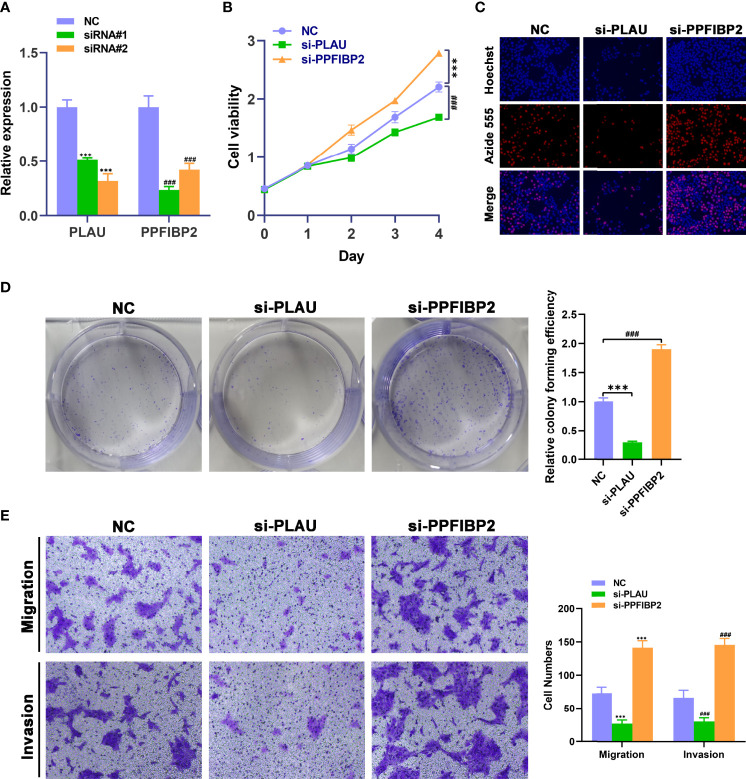
Biological functional validation of PLAU and PPFIBP2 in TSCC cells. **(A)** Silencing efficiency of PLAU and PPFIBP2 detected using qRT-PCR. CCK8 assay **(B)**, EDU assay **(C)**, and colony formation assay **(D)** showing the influence of PLAU and PPFIBP2 depletion on proliferation of Cal-27 cells. **(E)** Transwell assay was used to examine the effects of PLAU and PPFIBP2 silence on migration and invasion ability of Cal-27 cells (*p < 0.05, **p < 0.01, and ***p < 0.001, *n* = 3).

We further investigated whether these mRNAs promoted the development and progression of TSCC through *in vitro*. We selected *PLAU* and *PPFIBP2* after a careful literature review revealed that they have not been or rarely been studied in TSCC. The analysis sought to further verify the reliability and accuracy of our diagnostic model. Subsequently, siRNAs targeting *PLAU* and *PPFIBP2* were designed. Compared with NC-transfected Cal-27 cells, the expression level of *PLAU* or *PPFIBP2* was lower in cells transfected with si-PLAU-2 (named si-PLAU) or si-PPFIBP2-1 (named si-PPFIBP2) (**Figure 8A**). CCK8 and EdU assays showed that silencing *PLAU* significantly suppressed the proliferation of TSCC cells, whereas depletion of *PPFIBP2* could promote the growth of TSCC cells (**Figures 8B, C**). Similarly, the proliferative capacities of TSCC cells were markedly suppressed after knockdown of *PLAU* and strikingly enhanced after depletion of *PPFIBP2* by performing clone formation (**Figure 8D**), indicating that *PLAU* and *PPFIBP2* play important roles in the growth. Finally, Transwell assay findings showed that the depletion of *PLAU* markedly attenuated the migration and invasion ability of TSCC cells, while knockdown of *PPFIBP2* significantly enhanced the migration and invasion ability of TSCC cells (**Figure 8E**).

## Discussion

TSCC, which arises from the base of the tongue, is characterized by an aggressive biological behavior and heterogeneous survival. Even in patients with early-stage disease, tumor recurrence remains the main factor for the poor prognosis of TSCC, with a mortality rate of up to 87% (13). Currently, surgery combined with chemo-radiotherapy is a crucial treatment for TSCC. However, its therapeutic effects are far from satisfactory (14). Two recent randomized phase 3 trials showed that the supplementary treatment regime of induction chemotherapy did not provide a survival benefit when compared with concurrent chemo-radiotherapy (15, 16). The addition of cetuximab to platinum/5-fluorouracil chemotherapy conferred limited benefits, with the median OS improved from 7.4 to 10.1 months, albeit at the expense of serious toxicity (17). After the failure of first-line treatment, there is no effective drug to improve survival or quality of life of patients with TSCC. In the Keynote-012 phase 1b trial, immune checkpoint inhibitors achieved a longer durable response (18, 19). The subsequent phase 2 Keynote-055 study demonstrated that immunological treatments may improve survival in patients with platinum resistance (20). Multiple phase 3 randomized trials, including Checkmate 141, Keynote-040, and Keynote-048, all achieved satisfactory efficacy, especially for patients with PD-L1 tumor proportion score (percentage of tumor cells with membranous PD-L1 expression) ≥50% or PD-L1 combined score (CPS) ≥1% (21–23). Despite recent advances in immune-related clinical trials, the genomics and immune landscapes of TSCC are unclear.

In the present study, we accurately selected many prognostic genes and established clusters with different survival rates using a series of screening methods, including WGCNA and LASSO analysis. Cao et al. identified a long noncoding RNA (lncRNA) prognostic signature model using orthogonal partial least squares discrimination analysis (OPLS-DA) (24). Compared to the WGCNA, OPLS-DA was an improvement of the PLS-DA approach to discriminate between the two classes. OPLS-DA may filter out irrelevant information for classification and improve the predictive ability and effectiveness of the model (25). The purposes of WGCNA are to enroll genes with similar expression and to visualize the relationship between co-expression and clinical features. Thus, in this study, we chose WGCNA as the first step in selecting co-expressed genes.

Consensus clustering divided the samples into two clusters. We failed to find any differences in clinical features between the two clusters, except for survival status (**Supplementary Figure 4**). Interestingly, immune-related results suggested that these clusters may mediate the distribution of CD8+, CD4+, and follicular helper T cells and Tregs to alter the immune landscape of TSCC. Cancer immunotherapy aims to promote tumor-specific T-cell responses (26). CD8+ cytotoxic T lymphocytes are the preferred tool and key immune cells for killing tumors because they detect intracellular antigens that are presented by MHC class I molecules (27). CD8+ T cells contribute to adaptive immunity, although the development of this immunity is slower than the innate immunity mediated by natural killer cells and dendritic cells (28). Some studies have indicated that CD8+ T-cell infiltrates can improve the prognosis of patients with human papilloma virus (HPV)-positive or HPV-negative oropharyngeal and tonsillar cancers (29–32). For TSCC, we found that clusters with CD8+ T-cell infiltrates had significantly better survival, suggesting that CD8+ T cells may play an important role in the prediction of survival and alteration of the immune environment. In healthy individuals, T memory cells constitute only 2%–3% of the T cells. This type of T cell readily proliferates and can change all memory cell subgroups. The present findings indicate that memory CD4+ T cells may be associated with poor survival. This mechanism needs to be further investigated (33, 34). T follicular helper (Tfh) cells can induce humoral alloimmunity by helping naïve B cells differentiate into memory B cells and alloantibody-producing plasma cells within germinal centers (35). In addition, Tfh cells can function as major biomarkers to tailor immunosuppression for individualized therapy after transplantation. Recently, the immune signature of Tfh cells was shown to be an independent prognostic signature for OS in breast cancer (36) and lung cancer (37). Interestingly, we also observed that Tfh cells may correlate with better OS in TSCC. Tregs are a subset of immunosuppressive CD4+ T cells. HPV-positive oropharyngeal cancers contain abundant Tregs, which account for the lower CD8+/Treg ratios (38). In TSCC, it remains to be seen whether Tregs change the immune landscape owing to their limited distribution. Therefore, in the present study, we comprehensively demonstrated a specific tumor immune environment.

The *PLAU* and *PPFIBP2* hub genes were identified as effective prognostic biomarkers for OS and PFS. *PLAU* codes a serine protease and then promotes a proteolytic cascade to convert these proteases into their active forms (39, 40). *PLAU* is involved in tumor cell migration and invasion in pancreatic ductal adenocarcinoma and colorectal cancer (41, 42). In the present study, *PLAU* was upregulated in tumor tissues and was related to OS in the cohort of TCGA and GEO datasets. Results from the HPA dataset indicate that *PLAU* may induce tumorigenesis and metastasis (**Supplementary Figures 5C, D**). However, this gene is not associated with tumor mutational burden or microsatellite instability (**Supplementary Figure 5A**). *PPFIBP2* is a novel gene in the LAR protein-tyrosine phosphatase-interacting protein (liprin) family that has been reported to be an independent prognostic biomarker in prostate cancer and thyroid cancer (43, 44). These genes may also have protective roles in TSCC. Moreover, the biological role of *PPFIBP2* and the underlying mechanisms remain unclear. In the HPA datasets, *PPFIBP2* may have the ability to inhibit tumorigenesis (**Supplementary Figure 5E**). By utilizing pan-cancer analysis, we found that microsatellite instability was inversely correlated with *PPFIBP2*, which further clarifies the innate immune-related mechanism in TSCC (**Supplementary Figure 5B**). To verify the reliability of the prognostic biomarkers, we performed a series of experiments by knocking down *PLAU* and *PPFIBP2* in TSCC cells. Depletion of *PLAU* significantly inhibited the proliferation and weakened the invasiveness and migration of TSCC cells. The downregulation of *PPFIBP2* markedly promoted growth and enhanced migration and invasion of TSCC cells, implicating *PLAU* and *PPFIBP2* as prognostic biomarkers and therapeutic targets for TSCC.

## Conclusions

Major prognostic biomarkers were filtered using WGCNA, and different clusters were assembled. After analyzing the correlation among clusters, we comprehensively profiled the immune landscape and immune cell infiltration in the tumor environment. Furthermore, a TCGA database risk model was established. The model and crucial genes were verified using GEO databases. The *PLAU* and *PPFIBP2* hub genes were identified and validated *in vitro*.

## Data Availability Statement

The datasets presented in this study can be found in online repositories. The names of the repository/repositories and accession number(s) can be found in the article/**Supplementary Material**.

## Author Contributions

HW, XW, and YJ designed the study. YJ analyzed and interpreted the data, and wrote the original draft. ZW wrote this manuscript. WT and ML edited and revised the manuscript. All authors have seen and approved the final version of the manuscript.

## Funding

This work was supported by the Key Laboratory of Translational Radiation Oncology, Hunan Province (No. 2015TP1009), the Provincial Key Research and Development Program of Hunan Province (No. 2018SK2123), and Hunan Cancer Hospital Climb Plan (No. QH201905).

## Conflict of Interest

The authors declare that the research was conducted in the absence of any commercial or financial relationships that could be construed as a potential conflict of interest.

## Publisher’s Note

All claims expressed in this article are solely those of the authors and do not necessarily represent those of their affiliated organizations, or those of the publisher, the editors and the reviewers. Any product that may be evaluated in this article, or claim that may be made by its manufacturer, is not guaranteed or endorsed by the publisher.

## Supplementary Material

Supplementary Figure 1 Kaplan–Meier plot of 11 crucial genes in TCGA dataset.

Supplementary Figure 2 Kaplan–Meier plot of *IL34*, *INTS8*, *KDELR2*, and *LMF* in GEO datasets. **(A–C)** Kaplan–Meier plot of four genes in GSE31056 **(A)**, GSE41613 **(B)**, and GSE65858 **(C)**.

Supplementary Figure 3 Kaplan–Meier plot of *MAP7D3*, *OAF*, *PLXNB1*, and *ZFP3* in GEO datasets. **(A–C)** Kaplan–Meier plot of four genes in GSE31056 **(A)**, GSE41613 **(B)**, and GSE65858 **(C)**.

Supplementary Figure 4 Differences in clinical features in the two clusters.

Supplementary Figure 5 Hub genes supplemental data. **(A)** Correlation of *PLAU* with MSI and TMB. **(B)** Correlation of *PPFIBP2* with MSI and TMB. **(C)** Expression of *PLAU* in non-metastatic and metastatic tissue from immunohistochemistry. **(D)** Expression of *PLAU* in normal and cancer tissue based on immunohistochemistry. **(E)** Expression of *PPFIBP2* in normal and cancer tissue based on immunohistochemistry.

Supplementary Figure 6 Pan-cancer analysis of *PLAU*. **(A)** Differential expression of *PLAU* in cancer and normal tissue. **(B)** Correlation of *PLAU* and immune cells.

Supplementary Figure 7 Pan-cancer analysis of *PPFIBP2*. **(A)** Differential expression of *PPFIBP2* in cancer and normal tissue. **(B)** Correlation of *PPFIBP2* and immune cells.

Supplementary Table 1 Cox analysis of prognostic genes in TCGA.

Supplementary Table 2 The different expressed genes in TCGA in comparison of cancer and normal tissue.

Supplementary Table 3 The interacted genes from Cox analysis and the comparison of cancer and normal tissue.

Supplementary Table 4 Cox analysis of prognostic genes in GSE65858.

Supplementary Table 5 The interacted genes from turquoise co-expression module, TCGA and GSE65858 datasets.

Supplementary Table 6 The Coefficient of risk model based on the Lasso Cox regression.

## Abbreviations

TSCC, Tongue squamous cell carcinoma. PD-1, Programmed cell death 1. PD-L1, Programmed cell death ligand 1. WGCNA, Weighted gene co-expression network analysis. TCGA, The Cancer Genome Atlas. DEGs, Differentially expressed genes. OS, Overall survival. GO, Gene Oncology. ROC, Receiver operating characteristic.

## References

[1] Bello IO Soini Y Salo T. Prognostic Evaluation of Oral Tongue Cancer: Means, Markers and Perspectives (II). Oral Oncol (2010) 46(9):636–43. doi: 10.1016/j.oraloncology.2010.06.008 20637679

[2] Patel SC Carpenter WR Tyree S Couch ME Weissler M Hackman T. Increasing Incidence of Oral Tongue Squamous Cell Carcinoma in Young White Women, Age 18 to 44 Years. J Clin Oncol (2011) 29(11):1488–94. doi: 10.1200/JCO.2010.31.7883 21383286

[3] Mithani SK Mydlarz WK Grumbine FL Smith IM Califano JA. Molecular Genetics of Premalignant Oral Lesions. Oral Dis (2007) 13(2):126–33. doi: 10.1111/j.1601-0825.2006.01349.x 17305612

[4] Brenner H. Long-Term Survival Rates of Cancer Patients Achieved by the End of the 20th Century: A Period Analysis. Lancet (2002) 360(9340):1131–5. doi: 10.1016/S0140-6736(02)11199-8 12387961

[5] Scognamiglio T Chen YT. Beyond the Percentages of PD-L1-Positive Tumor Cells: Induced Versus Constitutive PD-L1 Expression in Primary and Metastatic Head and Neck Squamous Cell Carcinoma. Head Neck Pathol (2018) 12(2):221–9. doi: 10.1007/s12105-017-0857-3 PMC5953879 28948509

[6] Ferris RL Licitra L Fayette J Even C Blumenschein G Jr Harrington KJ. Nivolumab in Patients With Recurrent or Metastatic Squamous Cell Carcinoma of the Head and Neck: Efficacy and Safety in CheckMate 141 by Prior Cetuximab Use. Clin Cancer Res (2019) 25(17):5221–30. doi: 10.1158/1078-0432.CCR-18-3944 PMC7721346 31239321

[7] Schoenfeld JD Hanna GJ Jo VY Rawal B Chen YH Catalano PS. Neoadjuvant Nivolumab or Nivolumab Plus Ipilimumab in Untreated Oral Cavity Squamous Cell Carcinoma: A Phase 2 Open-Label Randomized Clinical Trial. JAMA Oncol (2020) 6(10):1563–70. doi: 10.1001/jamaoncol.2020.2955 PMC7453348 32852531

[8] Hussein AA Forouzanfar T Bloemena E de Visscher J Brakenhoff RH Leemans CR. A Review of the Most Promising Biomarkers for Early Diagnosis and Prognosis Prediction of Tongue Squamous Cell Carcinoma. Br J Cancer (2018) 119(6):724–36. doi: 10.1038/s41416-018-0233-4 PMC6173763 30131545

[9] Langfelder P Horvath S. WGCNA: An R Package for Weighted Correlation Network Analysis. BMC Bioinf (2008) 9:559. doi: 10.1186/1471-2105-9-559 PMC2631488 19114008

[10] Huang H Zhang Q Ye C Lv JM Liu X Chen L. Identification of Prognostic Markers of High Grade Prostate Cancer Through an Integrated Bioinformatics Approach. J Cancer Res Clin Oncol (2017) 143(12):2571–79. doi: 10.1007/s00432-017-2497-0 PMC11818995 28849390

[11] Niemira M Collin F Szalkowska A Bielska A Chwialkowska K Reszec J. Molecular Signature of Subtypes of Non-Small-Cell Lung Cancer by Large-Scale Transcriptional Profiling: Identification of Key Modules and Genes by Weighted Gene Co-Expression Network Analysis (WGCNA). Cancers (Basel) (2019) 12(1):37. doi: 10.3390/cancers12010037 PMC7017323 31877723

[12] Tang J Kong D Cui Q Wang K Zhang D Gong Y. Prognostic Genes of Breast Cancer Identified by Gene Co-Expression Network Analysis. Front Oncol (2018) 8:374. doi: 10.3389/fonc.2018.00374 30254986 PMC6141856

[13] Liao CT Chang JT Wang HM Ng SH Hsueh C Lee LY. Salvage Therapy in Relapsed Squamous Cell Carcinoma of the Oral Cavity: How and When? Cancer (2008) 112(1):94–103. doi: 10.1002/cncr.23142 18022917

[14] Montero PH Patel SG. Cancer of the Oral Cavity. Surg Oncol Clin N Am (2015) 24(3):491–508. doi: 10.1016/j.soc.2015.03.006 25979396 PMC5018209

[15] Cohen EE Karrison TG Kocherginsky M Mueller J Egan R Huang CH. Phase III Randomized Trial of Induction Chemotherapy in Patients With N2 or N3 Locally Advanced Head and Neck Cancer. J Clin Oncol (2014) 32(25):2735–43. doi: 10.1200/JCO.2013.54.6309 PMC4876357 25049329

[16] Haddad R O'Neill A Rabinowits G Tishler R Khuri F Adkins D. Induction Chemotherapy Followed by Concurrent Chemoradiotherapy (Sequential Chemoradio- Therapy) Versus Concurrent Chemoradiotherapy Alone in Locally Advanced Head and Neck Cancer (PARADIGM): A Randomised Phase 3 Trial. Lancet Oncol (2013) 14(3):257–64. doi: 10.1016/S1470-2045(13)70011-1 23414589

[17] Vermorken JB Mesia R Rivera F Remenar E Kawecki A Rottey S. Platinum-Based Chemotherapy Plus Cetuximab in Head and Neck Cancer. N Engl J Med (2008) 359(11):1116–27. doi: 10.1056/NEJMoa0802656 18784101

[18] Seiwert TY Burtness B Mehra R Weiss J Berger R Eder JP. Safety and Clinical Activity of Pembrolizumab for Treatment of Recurrent or Metastatic Squamous Cell Carcinoma of the Head and Neck (KEYNOTE-012): An Open-Label, Multicentre, Phase 1b Trial. Lancet Oncol (2016) 17(7):956–65. doi: 10.1016/S1470-2045(16)30066-3 27247226

[19] Chow LQM Haddad R Gupta S Mahipal A Mehra R Tahara M. Antitumor Activity of Pembrolizumab in Biomarker-Unselected Patients With Recurrent and/or Metastatic Head and Neck Squamous Cell Carcinoma: Results From the Phase Ib KEYNOTE-012 Expansion Cohort. J Clin Oncol (2016) 34(32):3838–45. doi: 10.1200/JCO.2016.68.1478 PMC6804896 27646946

[20] Bauml J Seiwert TY Pfister DG Worden F Liu SV Gilbert J. Pembrolizumab for Platinum- and Cetuximab-Refractory Head and Neck Cancer: Results From a Single-Arm, Phase II Study. J Clin Oncol (2017) 35(14):1542–49. doi: 10.1200/JCO.2016.70.1524 PMC5946724 28328302

[21] Harrington KJ Ferris RL Blumenschein G Jr Colevas AD Fayette J Licitra L. Nivolumab Versus Standard, Single-Agent Therapy of Investigator's Choice in Recurrent or Metastatic Squamous Cell Carcinoma of the Head and Neck (CheckMate 141): Health-Related Quality-of-Life Results From a Randomised, Phase 3 Trial. Lancet Oncol (2017) 18(8):1104–15. doi: 10.1016/S1470-2045(17)30421-7 PMC6461049 28651929

[22] Pei R Shi Y Lv S Dai T Zhang F Liu S. Nivolumab vs Pembrolizumab for Treatment of US Patients With Platinum-Refractory Recurrent or Metastatic Head and Neck Squamous Cell Carcinoma: A Network Meta-Analysis and Cost- Effectiveness Analysis. JAMA Netw Open (2021) 4(5):e218065. doi: 10.1001/jamanetworkopen 33956130 PMC8103222

[23] Burtness B Harrington KJ Greil R Soulières D Tahara M de Castro G Jr. Pembrolizumab Alone or With Chemotherapy Versus Cetuximab With Chemotherapy for Recurrent or Metastatic Squamous Cell Carcinoma of the Head and Neck (KEYNOTE-048): A Randomised, Open-Label, Phase 3 Study. Lancet (2019) 394(10212):1915–28. doi: 10.1016/S0140-6736(19)32591-7 31679945

[24] Cao W Liu JN Liu Z Wang X Han ZG Ji T. A three-lncRNA Signature Derived From the Atlas of ncRNA in Cancer (TANRIC) Database Predicts the Survival of Patients With Head and Neck Squamous Cell Carcinoma. Oral Oncol (2017) 65:94–101. doi: 10.1016/j.oraloncology.2016.12.017 28109476

[25] Gu X Coates P Wang L Erdogan B Salehi A Sgaramella N. Variation in Plasma Levels of TRAF2 Protein During Development of Squamous Cell Carcinoma of the Oral Tongue. Front Oncol (2021) 11:753699. doi: 10.3389/fonc.2021.753699 34888239 PMC8649619

[26] Chen DS Mellman I. Oncology Meets Immunology: The Cancer-Immunity Cycle. Immunity (2013) 39(1):1–10. doi: 10.1016/j.immuni.2013.07.012 23890059

[27] Farhood B Najafi M Mortezaee K. CD8+ Cytotoxic T Lymphocytes in Cancer Immunotherapy: A Review. J Cell Physiol (2019) 234(6):8509–21. doi: 10.1002/jcp.27782 30520029

[28] Dranoff G. Cytokines in Cancer Pathogenesis and Cancer Therapy. Nat Rev Cancer (2004) 4(1):11–22. doi: 10.1038/nrc1252 14708024

[29] Ward MJ Thirdborough SM Mellows T Riley C Harris S Suchak K. Tumour- Infiltrating Lymphocytes Predict for Outcome in HPV-Positive Oropharyngeal Cancer. Br J Cancer (2014) 110(2):489–500. doi: 10.1038/bjc.2013.639 24169344 PMC3899750

[30] Oguejiofor K Hall J Slater C Betts G Hall G Slevin N. Stromal Infiltration of CD8 T Cells is Associated With Improved Clinical Outcome in HPV-Positive Oropharyngeal Squamous Carcinoma. Br J Cancer (2015) 113(6):886–93. doi: 10.1038/bjc.2015.277 PMC4578081 26313665

[31] Näsman A Romanitan M Nordfors C Grün N Johansson H Hammarstedt L. Tumor Infiltrating CD8+ and Foxp3+ Lymphocytes Correlate to Clinical Outcome and Human Papillomavirus (HPV) Status in Tonsillar Cancer. PloS One (2012) 7(6):e38711. doi: 10.1371/journal.pone.0038711 22701698 PMC3373553

[32] Matlung SE Wilhelmina van Kempen PM Bovenschen N van Baarle D Willems SM. Differences in T-Cell Infiltrates and Survival Between HPV+ and HPV- Oropharyngeal Squamous Cell Carcinoma. Future Sci OA (2016) 2(1):FSO88. doi: 10.4155/fso.15.88 28031938 PMC5137981

[33] Vahidi Y Faghih Z Talei AR Doroudchi M Ghaderi A. Memory CD4+ T Cell Subsets in Tumor Draining Lymph Nodes of Breast Cancer Patients: A Focus on T Stem Cell Memory Cells. Cell Oncol (Dordr) (2018) 41(1):1–11. doi: 10.1007/s13402-017-0352-6 28994018 PMC12995230

[34] Becattini S Latorre D Mele F Foglierini M De Gregorio C Cassotta A. T Cell Immunity. Functional Heterogeneity of Human Memory CD4^+^ T Cell Clones Primed by Pathogens or Vaccines. Science (2015) 347(6220):400–6. doi: 10.1126/science.1260668 25477212

[35] Yan L de Leur K Hendriks RW van der Laan LJW Shi Y Wang L. T Follicular Helper Cells As a New Target for Immunosuppressive Therapies. Front Immunol (2017) 8:1510. doi: 10.3389/fimmu.2017.01510 29163552 PMC5681999

[36] Gu-Trantien C Loi S Garaud S Equeter C Libin M de Wind A. CD4^+^; Follicular Helper T Cell Infiltration Predicts Breast Cancer Survival. J Clin Invest (2013) 123(7):2873–92. doi: 10.1172/JCI67428 PMC3696556 23778140

[37] Xu F Zhang H Chen J Lin L Chen Y. Immune Signature of T Follicular Helper Cells Predicts Clinical Prognostic and Therapeutic Impact in Lung Squamous Cell Carcinoma. Int Immunopharmacol (2020) 81:105932. doi: 10.1016/j.intimp.2019.105932 31836430

[38] Mandal R Şenbabaoğlu Y Desrichard A Havel JJ Dalin MG Riaz N. The Head and Neck Cancer Immune Landscape and its Immunotherapeutic Implications. JCI Insight (2016) 1(17):e89829. doi: 10.1172/jci.insight.89829 27777979 PMC5070962

[39] Montuori N Pesapane A Rossi FW Giudice V De Paulis A Selleri C. Urokinase Type Plasminogen Activator Receptor (uPAR) as a New Therapeutic Target in Cancer. Transl Med UniSa (2016) 15:15–21. doi: 10.1186/s12967-022-03329-3 27896223 PMC5120746

[40] Mahmood N Mihalcioiu C Rabbani SA. Multifaceted Role of the Urokinase-Type Plasminogen Activator (uPA) and Its Receptor (uPAR): Diagnostic, Prognostic, and Therapeutic Applications. Front Oncol (2018) 8:24. doi: 10.3389/fonc.2018.00024 29484286 PMC5816037

[41] Chen Q Yu D Zhao Y Qiu J Xie Y Tao M. Screening and Identification of Hub Genes in Pancreatic Cancer by Integrated Bioinformatics Analysis. J Cell Biochem (2019) 120(12):19496–508. doi: 10.1002/jcb.29253 31297881

[42] Lin M Zhang Z Gao M Yu H Sheng H Huang J. MicroRNA-193a-3p Suppresses the Colorectal Cancer Cell Proliferation and Progression Through Downregulating the PLAU Expression. Cancer Manag Res (2019) 11:5353–63. doi: 10.2147/CMAR.S208233 PMC6578599 31354344

[43] Iyama K Matsuse M Mitsutake N Rogounovitch T Saenko V Suzuki K. Identification of Three Novel Fusion Oncogenes, SQSTM1/NTRK3, AFAP1L2/ RET, and PPFIBP2/RET, in Thyroid Cancers of Young Patients in Fukushima. Thyroid (2017) 27(6):811–8. doi: 10.1089/thy.2016.0673 28351223

[44] Wu Y Yu H Zheng SL Feng B Kapron AL Na R. Germline Mutations in PPFIBP2 are Associated With Lethal Prostate Cancer. Prostate (2018) 78(16):1222– 28. doi: 10.1002/pros.23697 30043417

