# Supplementary figures and images for: An Integrated Analysis of Prognostic Signature and Immune Microenvironment in Tongue Squamous Cell Carcinoma
Source: Front Oncol. 2022 Jul 13;12:891716. doi: 10.3389/fonc.2022.891716 (PMC9326056; doi:10.3389/fonc.2022.891716)

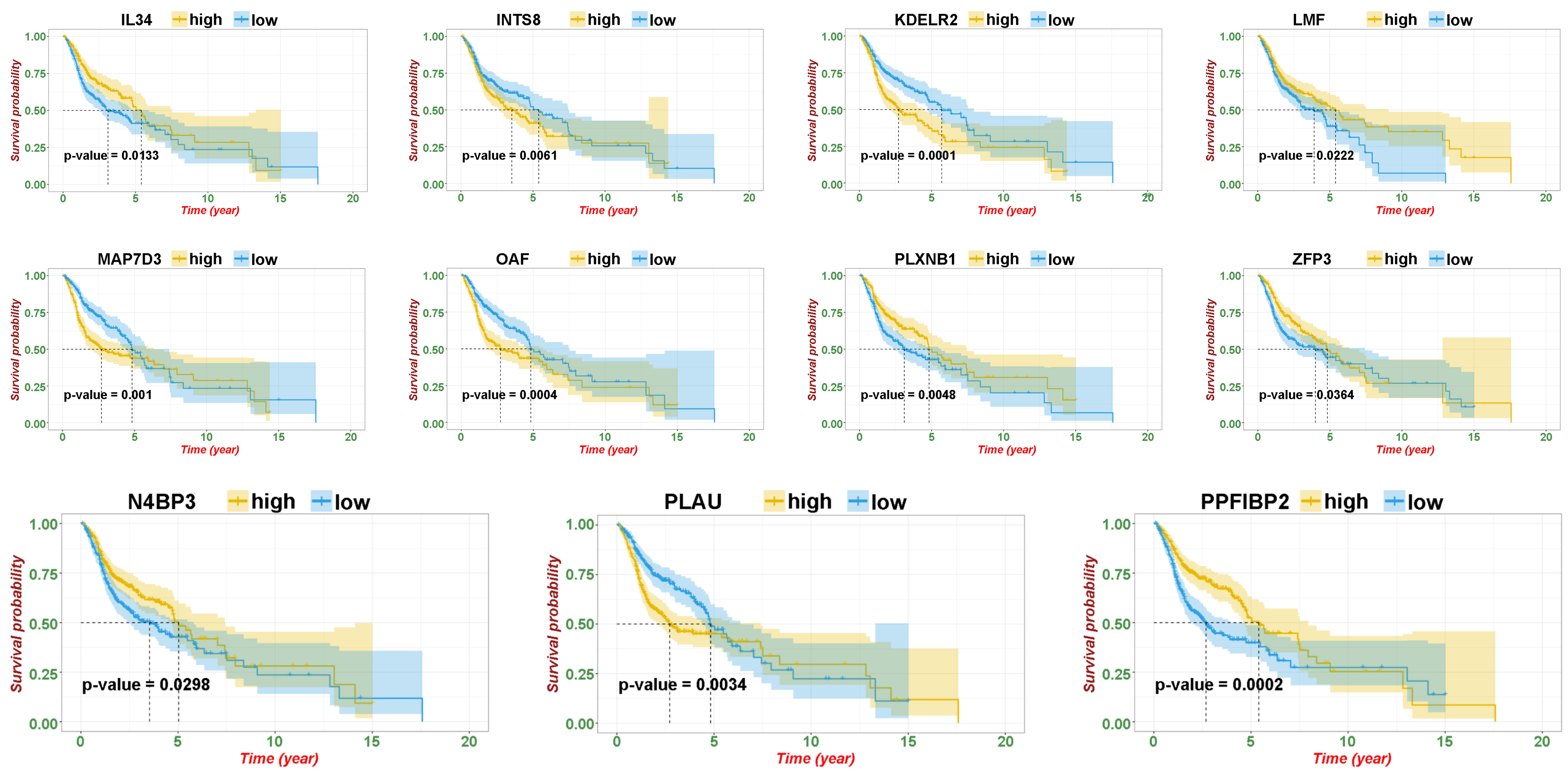

Supplement: Supplementary Figure 1 — Kaplan–Meier plot of 11 crucial genes in TCGA dataset. [file Image_1.tif]

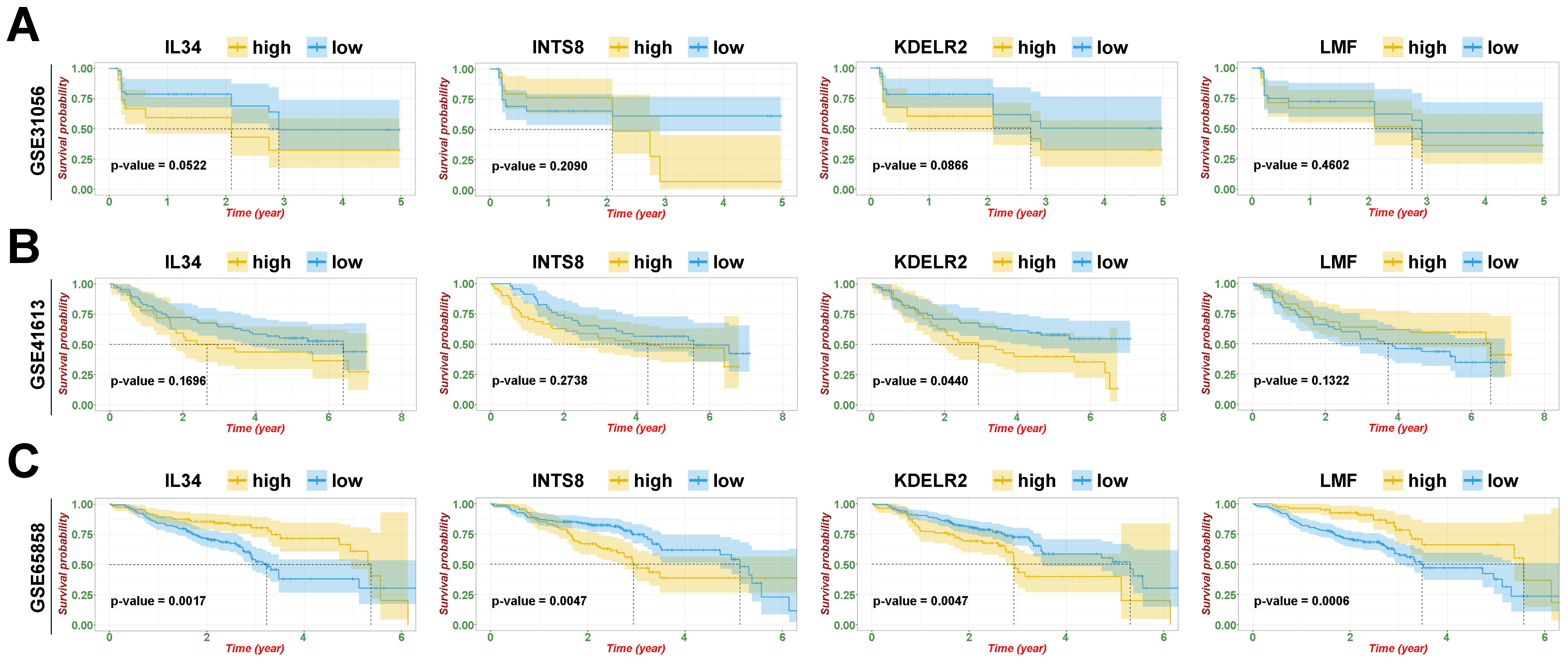

Supplement: Supplementary Figure 2 — Kaplan–Meier plot of IL34, INTS8, KDELR2, and LMF in GEO datasets. (A–C) Kaplan–Meier plot of four genes in GSE31056 (A), GSE41613 (B), and GSE65858 (C). [file Image_2.tif]

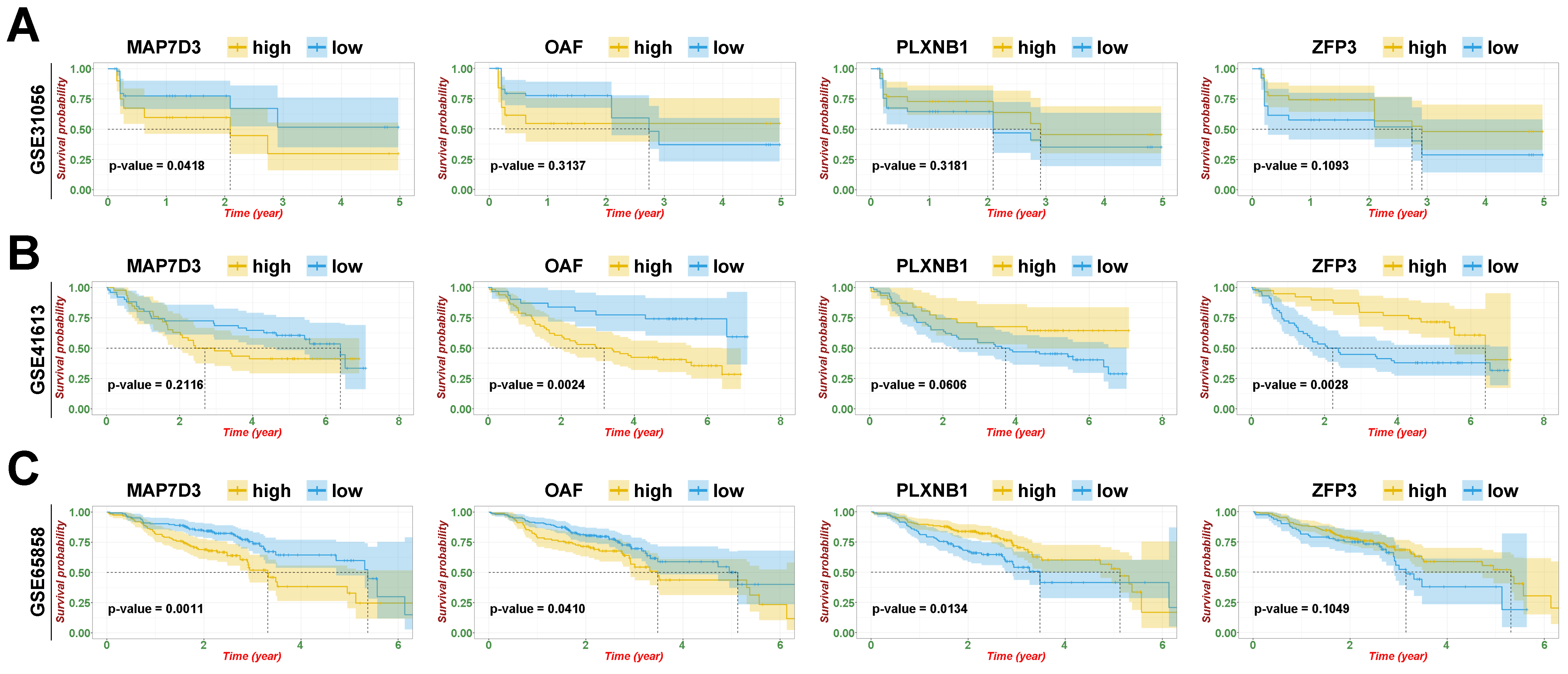

Supplement: Supplementary Figure 3 — Kaplan–Meier plot of MAP7D3, OAF, PLXNB1, and ZFP3 in GEO datasets. (A–C) Kaplan–Meier plot of four genes in GSE31056 (A), GSE41613 (B), and GSE65858 (C). [file Image_3.tif]

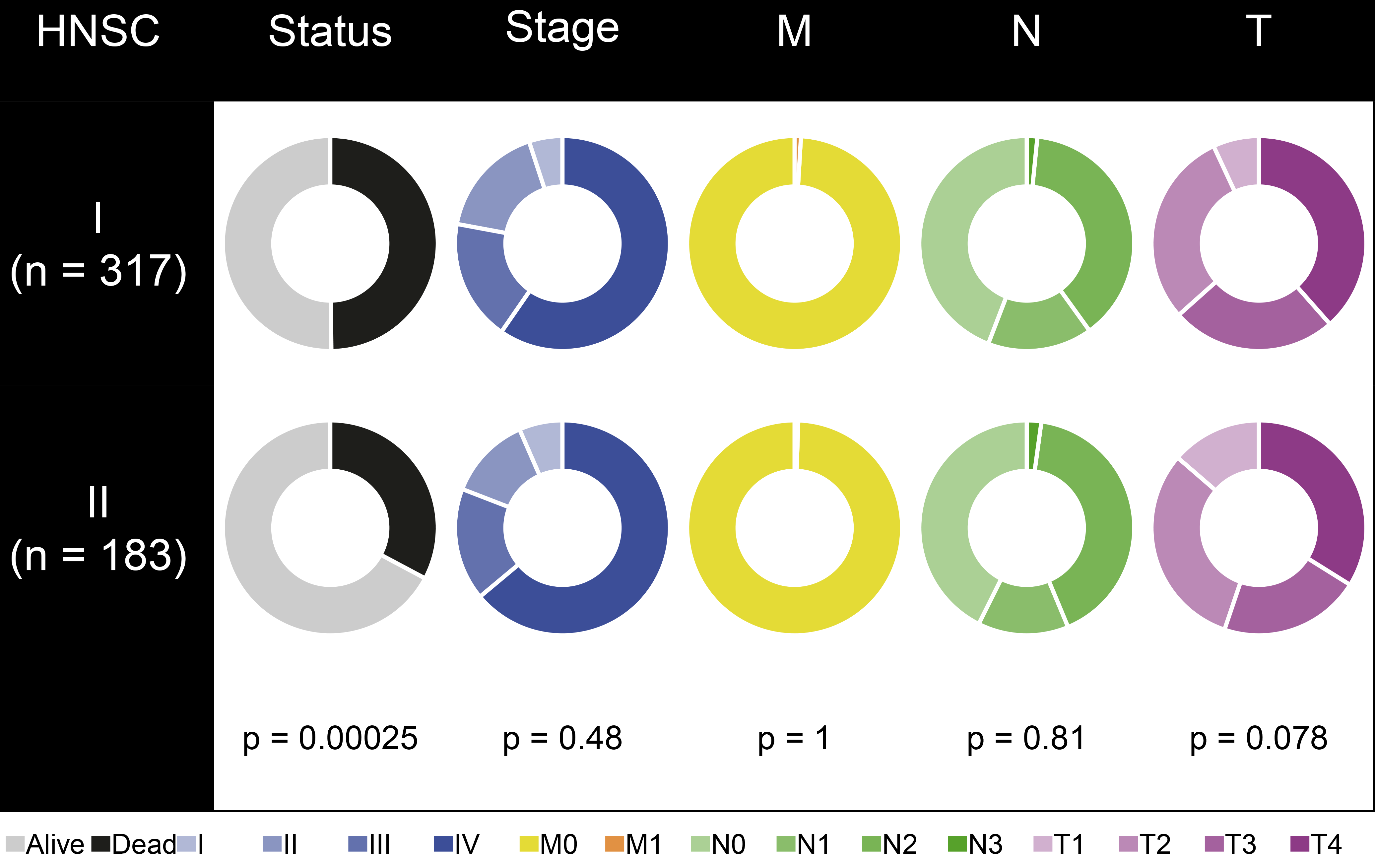

Supplement: Supplementary Figure 4 — Differences in clinical features in the two clusters. [file Image_4.tif]

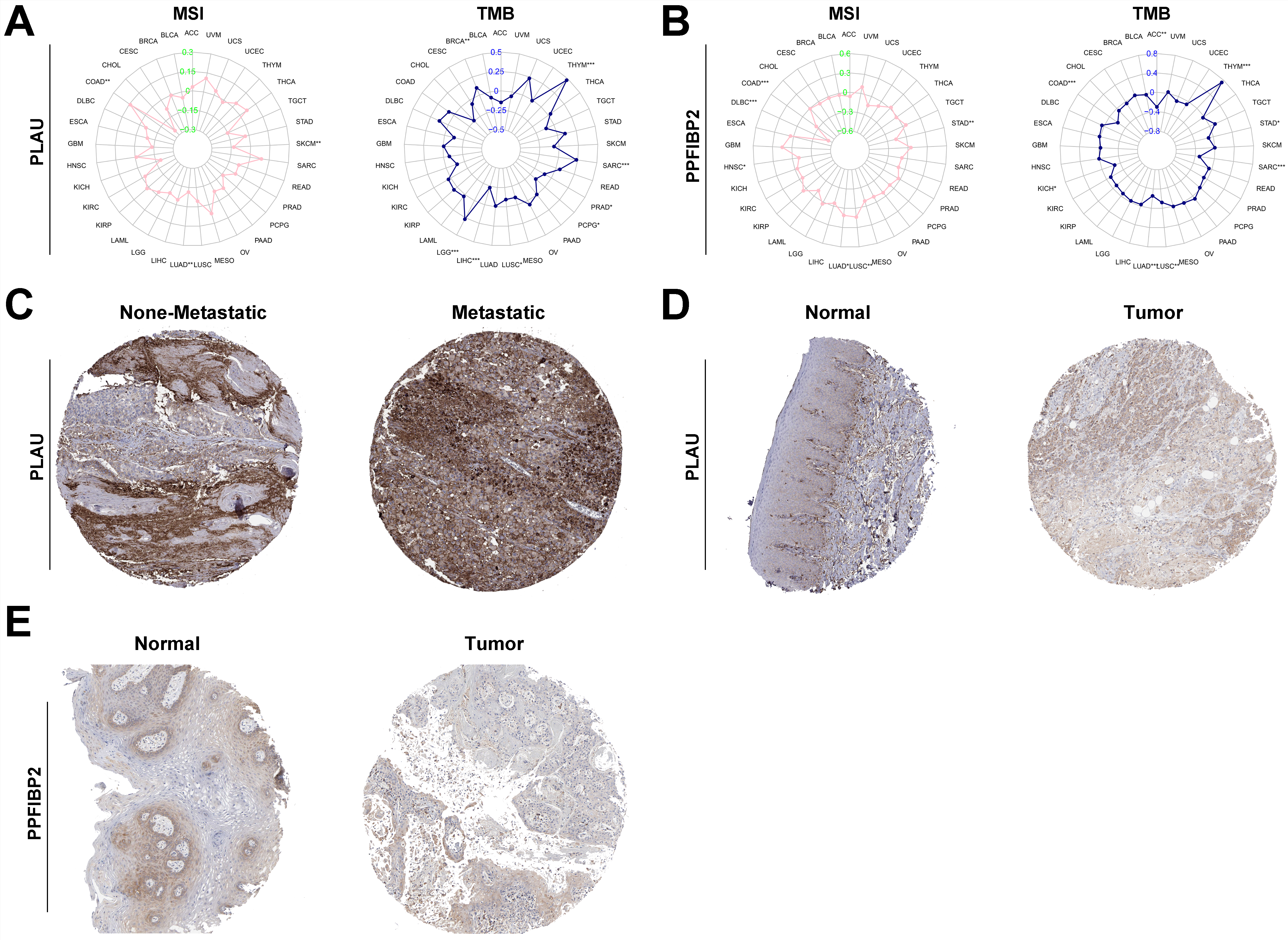

Supplement: Supplementary Figure 5 — Hub genes supplemental data. (A) Correlation of PLAU with MSI and TMB. (B) Correlation of PPFIBP2 with MSI and TMB. (C) Expression of PLAU in non-metastatic and metastatic tissue from immunohistochemistry. (D) Expression of PLAU in normal and cancer tissue based on immunohistochemistry. (E) Expression of PPFIBP2 in normal and cancer tissue based on immunohistochemistry. [file Image_5.tif]

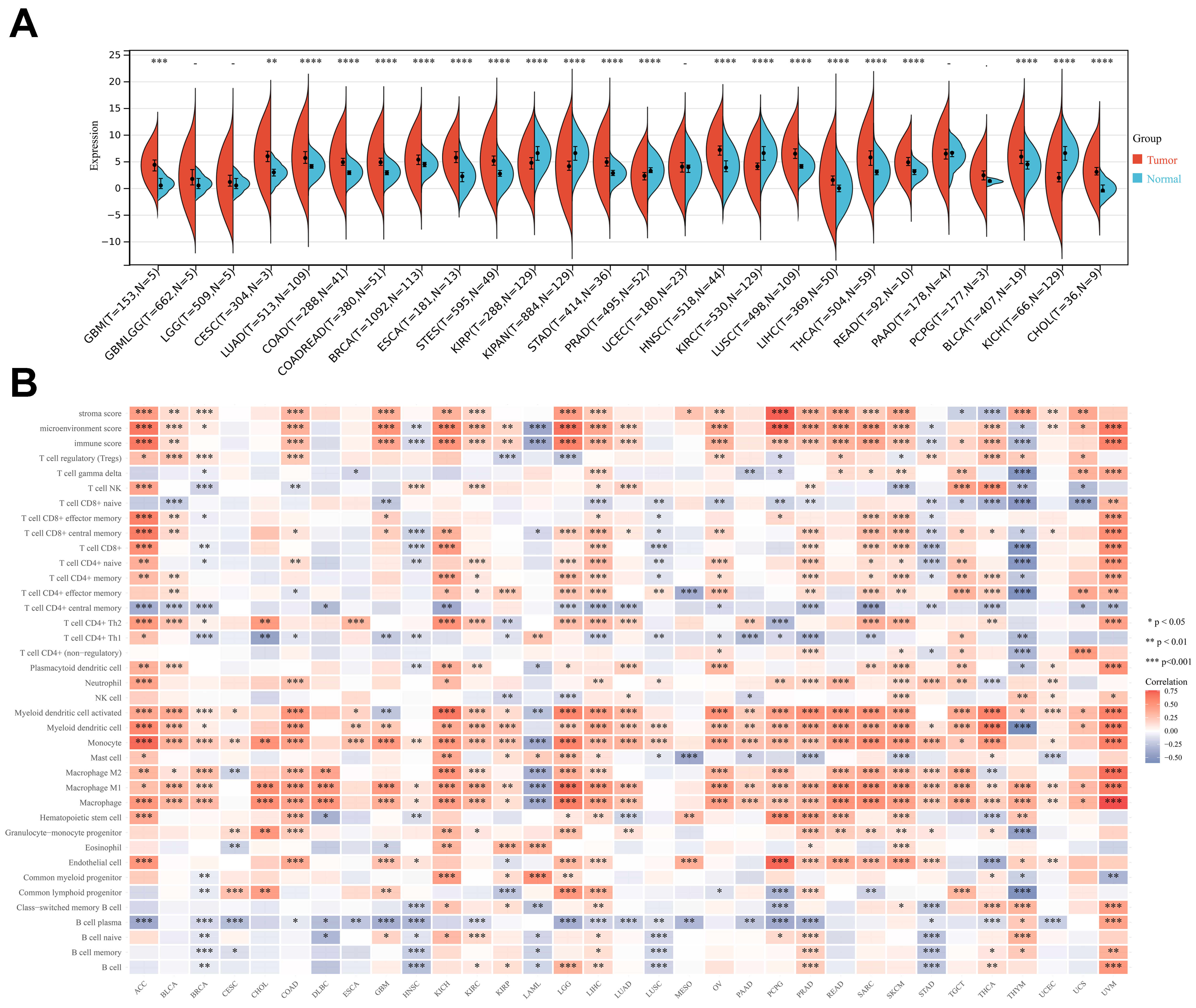

Supplement: Supplementary Figure 6 — Pan-cancer analysis of PLAU. (A) Differential expression of PLAU in cancer and normal tissue. (B) Correlation of PLAU and immune cells. [file Image_6.tif]

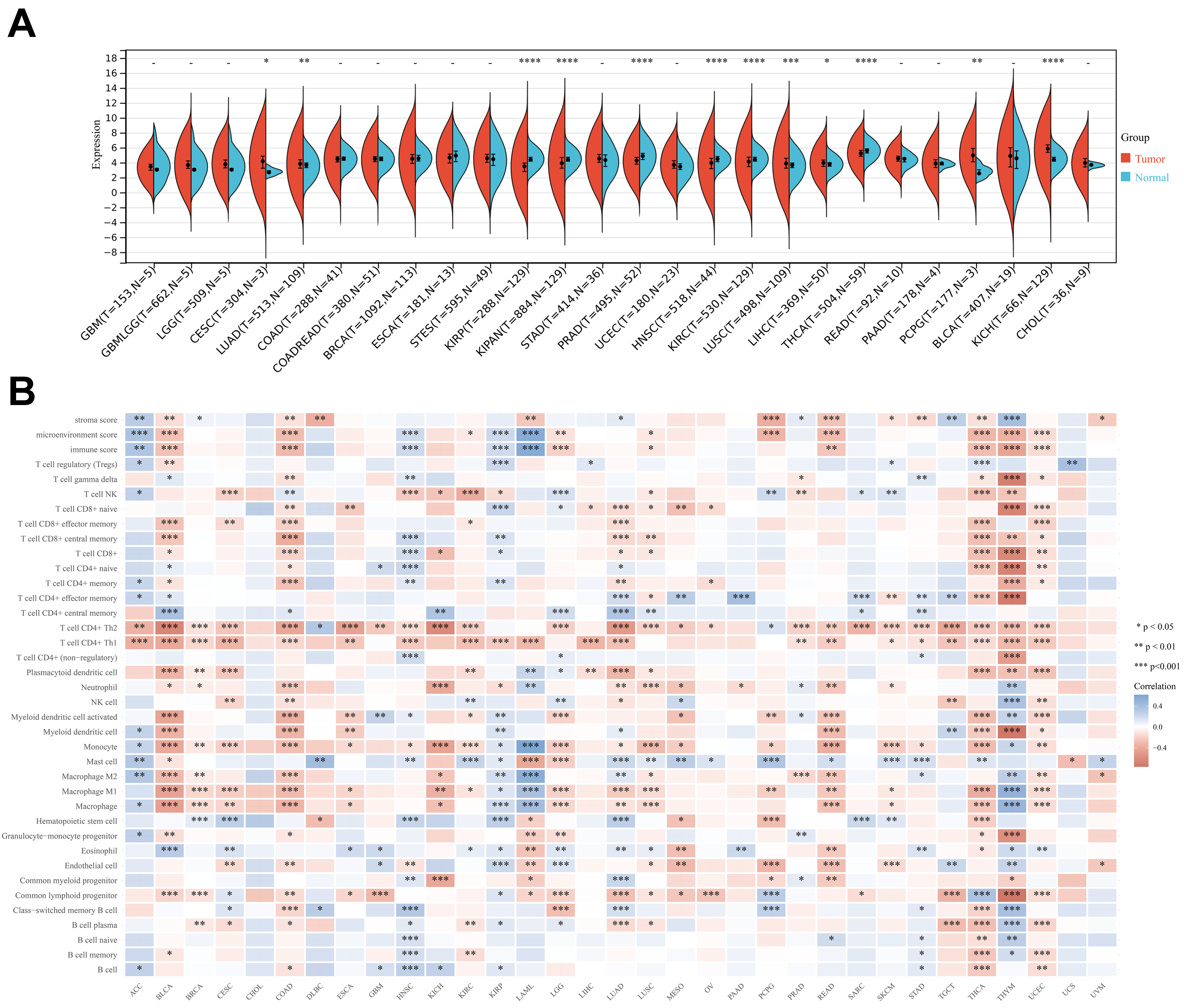

Supplement: Supplementary Figure 7 — Pan-cancer analysis of PPFIBP2. (A) Differential expression of PPFIBP2 in cancer and normal tissue. (B) Correlation of PPFIBP2 and immune cells. [file Image_7.tif]
